# Supplementary material for: Probing plexciton emission from 2D materials on gold nanotrenches
Source: Nat Commun. 2024 Nov 6;15:9583. doi: 10.1038/s41467-024-53669-5 (PMC11541865; doi:10.1038/s41467-024-53669-5)
Supplement: Supplementary file 1 — Supplementary Info [file 41467_2024_53669_MOESM1_ESM.pdf]

SUPPLEMENTARY INFORMATION
Probing Plexciton Emission from 2D Materials on Gold Nanotrenches

Junze Zhou,[1] P. A. D. Gonçalves,[2] Fabrizio Riminucci,[1] Scott Dhuey,[1] Edward Barnard,[1] Adam Schwartzberg,[1] F. Javier García de Abajo,[2,3] and Alexander Weber-Bargioni[1]
[1] *The Molecular Foundry, Lawrence Berkeley National Laboratory, 1 Cyclotron Road, Berkeley, CA 94720, USA*
[2] *ICFO – Institut de Ciencies Fotoniques, The Barcelona Institute of Science and Technology, 08860 Castelldefels (Barcelona), Spain*
[3] *ICREA – Institució Catalana de Recerca i Estudis Avançats, Passeig Lluís Companys 23, 08010 Barcelona, Spain*

CONTENTS

S1. Sample preparation and dielectric function of WSe_2 2
A. Gold nanotrenches: Template stripping 2
B. WSe_2 monolayer: Exfoliation and dry transfer 2
C. Fit of the dielectric function of WSe_2 from reflection measurements 3
S2. Fiber-coupled scanning near-field optical microscope 3
S3. Theory: Optical response of gold nanotrenches 6
A. Modeling of plasmon modes in gold nanotrench using BEM 7
B. Plasmonic resonances in arrays of gold nanotrenches: Grating modes and channel resonances 8
C. Characterization of the plasmon resonance in a gold nanotrench 9
S4. Tuning the nanotrench plasmon resonance: Influence of ALD coating and trench shape 10
A. Experiment 10
B. Theory: Effect of the nanotrench shape 11
C. Influence of the ALD coating 13
D. Polarization dependence 14
E. Impact of PDMS residues 14
S5. Plasmon–exciton coupling in WSe_2-covered gold nanotrenches 16
A. Plasmon–exciton dispersion diagram and coupled-oscillator model fit 16
1. Influence of the uncoupled exciton background on the determination of the Rabi-like splitting 18
B. Spatial localization of the lower polariton 19
S6. Additional measurements 20
A. Reflection data from an off-resonance control sample and from a zero-detuned sample 20
B. Line scans on different plexciton samples 20
C. Lorentzian peak fitting and polarization dependence of the plexciton PL emission 20
Supplementary references 22

S1. SAMPLE PREPARATION AND DIELECTRIC FUNCTION OF WSe_2

A. Gold nanotrenches: Template stripping

The gold nanotrenches were fabricated by the template stripping method, where hydrogen silsesquioxane (HSQ) narrow lines serve as a template for the nanotrenches. The fabrication involved the following steps:

1. Spin coating of 2% HSQ on a polished silicon wafer using different spin speeds for achieving the target thicknesses (namely, 1000 rpm and 2000 rpm for 46 nm and 34 nm, respectively).
2. Exposing the negative resist by the electron-beam lithography using Raith EBPG 5200, creating narrow lines with specific widths. The width of the lines was controlled by varying the electron-beam dose (in the 25 000 – 76 476 $\mu C\,cm^{-2}$ range). For each dose, the designed width was in the 6 – 14 nm range. We wrote two arrays, with pitch sizes 680 nm and 1000 nm, using the same dose and designed width.
3. Development of the HSQ (using 4% NaCl in 1% NaOH for 4 min) to obtain the narrow lines that served as a template for the nanotrenches.
4. Electron-beam evaporation of a 120-nm-thick gold film on the templated sample.
5. Placement of a droplet of Ormocomp photoresist on top of the gold and covering it with a transparent substrate.
6. UV-light curing of the Ormocomp for 90 min.
7. Peeling the gold off the templated sample with a glass substrate.
8. Coating the top surface of the patterned gold substrate with approximately 2 nm of Al_2O_3 grown by atomic-layer deposition (ALD), which functioned as a spacer layer to reduce the Ohmic contact and photoluminescence (PL) quenching.

B. WSe_2 monolayer: Exfoliation and dry transfer

The WSe_2 monolayer was prepared using mechanical exfoliation. Initially, the WSe_2 crystal was thinned down by applying adhesive tape multiple times until the color of the crystal exhibited a reddish hue. The WSe_2 on the tape was subsequently exfoliated onto a polydimethylsiloxane (PDMS) substrate. The monolayer on PDMS was identified under an optical microscope with a $\times 20$ objective lens by analyzing variations in color contrast among the substrate, bulk structures, and monolayers. Once identified within the PDMS substrate, the monolayer was carefully transferred onto both unpatterned (flat) and patterned (with nanotrenches) substrates using the well-established polymer-stamp transfer process described in Ref. [1]. Figure S1a shows a transferred WSe_2 onto an unpatterned gold substrate. The monolayer is clearly identified by the bright emission signal in photoluminescence (PL), which matches the features in the corresponding camera view. Figure S1b confirms the spectral properties of the monolayer, with the PL spectrum centered at around 740 nm. The consistent intensities in different areas of the flake indicate the uniformity of the monolayer.

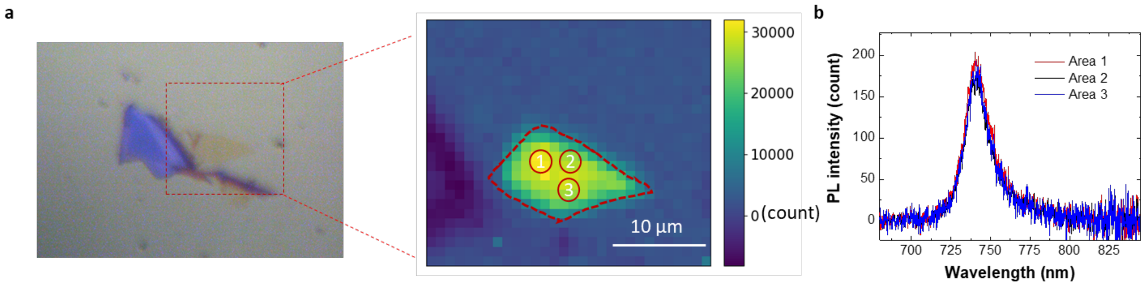

Fig. S1. **(a)** Camera view (left) and PL intensity image (right) of WSe_2 transferred onto an unpatterned gold substrate. The PL image is obtained by the integrating emission signal within the 725 – 775 nm wavelength range, and the bright emission region corresponds to the WSe_2 monolayer. **(b)** Photoluminescence spectra from the areas 1, 2, and 3 marked in **a**.

C. Fit of the dielectric function of WSe_2 from reflection measurements

We fitted the dielectric function of WSe_2 using the measured reflection from an unpatterned region of the sample (Fig. S2). More precisely, we used the ratio $\Delta R = R_{WSe_2+subs}/R_{subs}$, where R_{WSe_2+subs} is the reflection from a WSe_2-covered region of the sample and R_{subs} is the reflection from the substrate alone, the latter being composed of a ultrathin ($t_{ALD} \approx 2\,nm$) ALD-layer of Al_2O_3 on gold (Fig. S2).

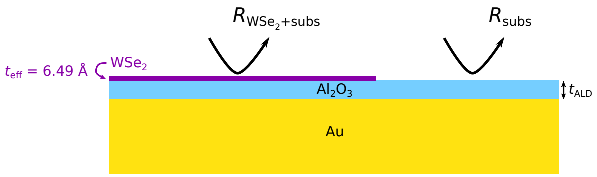

Fig. S2. Schematic representation of the unpatterned system (without the nanotrenches) used to retrieve the dielectric function of WSe_2.

We describe the WSe_2 monolayer as an atomically thin slab with an effective thickness $t_{eff} = 6.49$ Å (corresponding to the interlayer spacing in the bulk material [2, 3]) and model its complex dielectric function (in the spectral region of interest) as a superposition of two Lorentzian terms:

$$\epsilon_{WSe_2}(\omega) = \epsilon_b + \sum_{j=1}^{2} \frac{f_j\, \omega_j^2}{\omega_j^2 - \omega^2 - i\omega\gamma_j}, \tag{S1}$$

with the fitting parameters listed in Table S1 and the result of the fit shown in Fig. S3.

ϵ_b		f_j	$\hbar\omega_j$ (eV)	$\hbar\gamma_j$ (eV)
7	$j = 1$	0.493	1.683	0.0467
	$j = 2$	1.567	2	0.354

Table S1. Fitting parameters for the dielectric function of WSe_2 [see Eq. (S1)].

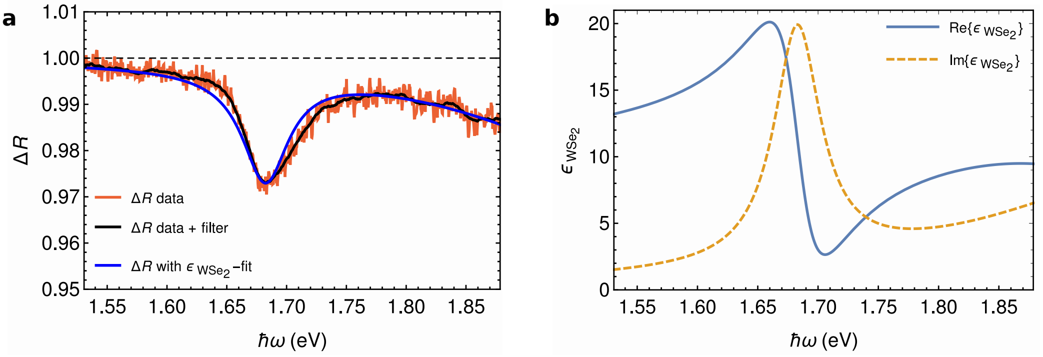

Fig. S3. Dielectric function of WSe_2 determined from reflection measurements. **(a)** Relative reflection, $\Delta R = R_{WSe_2+subs}/R_{subs}$, including the raw experimental data (red), the same data smoothed with a median filter [to facilitate the fitting procedure] (blue), and the result of the fit (blue). **(b)** Corresponding ϵ_{WSe_2} fit [see Eq. (S1) and Table S1].

S2. FIBER-COUPLED SCANNING NEAR-FIELD OPTICAL MICROSCOPE

The nanoimprinted probe was prepared by the method described in Ref. [4]. The PL measurement setup adopted a fiber-in–fiber-out configuration, as depicted in Fig. S4a. In this setup, the excitation laser was coupled to the

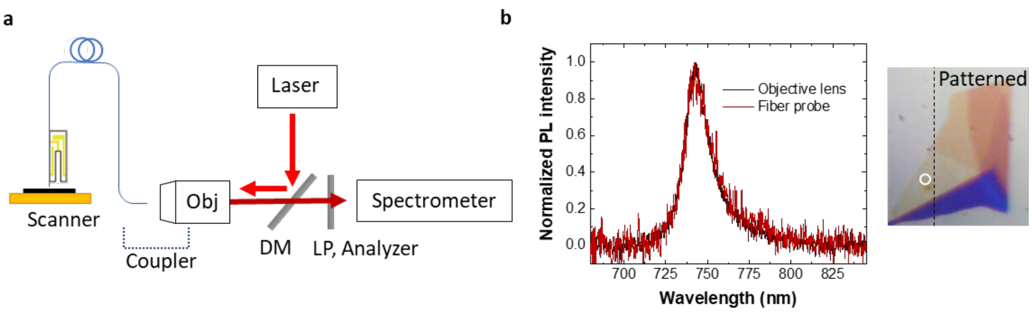

Fig. S4. (**a**) Schematic of the PL measurement setup using our fiber-based probe [Obj: objective lens, DM: dichroic mirror, LP: linear polarizer]. (**b**) Comparison of PL spectrum measured through an objective lens with that recorded using the near-field probe. Here, the PL emission was recorded from the unpatterned region, as marked in the left-side of the dashed line in the camera-view image.

fiber and the emitted signal under the probe was collected through the same path. We acquired a background spectrum in the area away from the WSe_2 monolayer, which allowed us to subtract the spectral response and the refractive-index dispersion of the fiber probe. To verify that this subtraction was accurate, we compared the PL spectrum obtained through the fiber probe with the PL spectrum collected by using an objective lens. The overlap of the PL spectra (Fig. S4b), recorded in the unpatterned gold region, confirmed the fidelity of the PL signal collected by our fiber-based near-field probe.

Hyperspectral maps were collected by recording the PL emission spectrum at each pixel. Figures S5a–b show PL emission maps obtained by scanning the sample and integrating the PL signal within the 650 – 800 nm range. Furthermore, using a linear polarizer (see Fig. S4a), we examined the polarization properties of the emitted PL: when the polarization was parallel to the nanotrenches it was difficult to resolve a significant emission contrast between the nanotrenches and the intermediate area (Fig. S5a); conversely, when the polarization was perpendicular to the nanotrenches, bright stripes with the periodicity of the fabricated trenches became readily apparent (Fig. S5b). These results indicate that the PL emission at the nanotrenches is linearly polarized (perpendicularly to them) and that it is substantially enhanced when compared with the PL signal coming from the flat region between the trenches. Figures S5c and S5d provide the correlated shear-force height map and the PL map, respectively, near a single nanotrench. Again, notice the enhancement of the PL emission signal at the nanotrench site. The decreased intensity observed in the bottom part of Fig. S5d corresponds to a region where the monolayer is spatially separated from the nanotrench, as evidenced by the observed height difference within the dotted contour in the shear-force height map depicted in Fig. S5c.

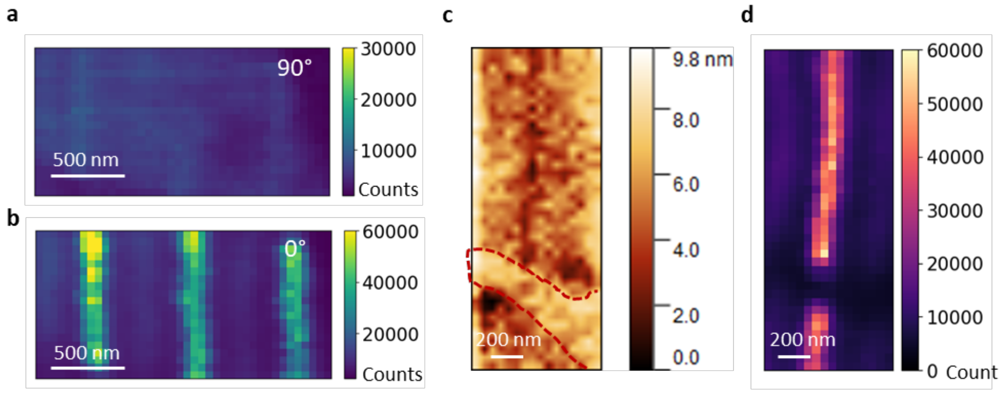

Fig. S5. Height and photoluminescence-intensity spatial maps. (**a**) and (**b**) correspond to PL maps (2 μm × 1 μm, 50 nm pixel size) for 0° polarization (parallel to the nanotrenches) and 90° polarization (perpendicular to the nanotrenches), respectively. The sample has nanotrenches with a period of P = 680 nm. (**c**) Shear-force height map. The red dotted contour highlights the area where the 2D layer has detached from the nanotrench. (**d**) Correlated PL map.

Figure S6 shows a larger area scan. In particular, in Fig. S6c, are large step size was used and so the trench positions are not visible in some lines. To provide a clearer depiction of the trench, Fig. S6b,d show data obtained using a cantilever-type sharp AFM probe (ATEC-NC), which show the trenches with higher resolution.

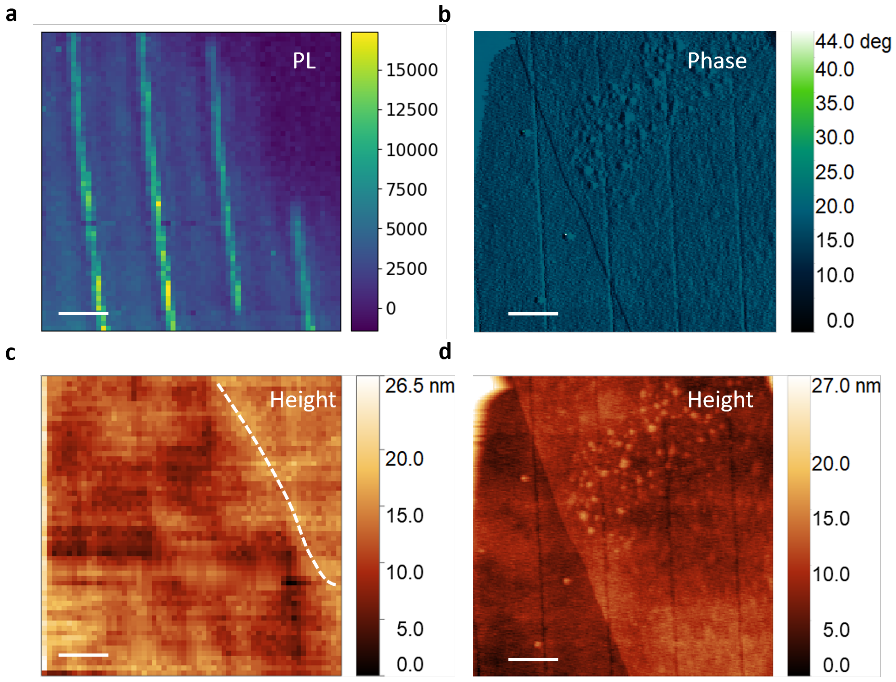

Fig. S6. Additional data, complementing that of Fig. S5. **(a,c)** Correlated PL and shear-force height image. The white dashed line indicates the edge of the monolayer. **(b,d)** Phase and height images recorded using a cantilever-type AFM probe (ATEC-NC). The scalebars are 500 nm.

S3. THEORY: OPTICAL RESPONSE OF GOLD NANOTRENCHES

We compute the optical response and the ensuing plasmon resonances associated with the gold nanotrenches using two different approaches: one considering a one-dimensional (1D) grating structure (Fig. S7a), and another assuming an isolated nanotrench (Fig. S7b). In the former, we calculate the reflectance of a plane wave from the grating using the rigorous coupled-wave analysis (RCWA) technique [5, 6, 7], while in the latter we determine the dispersion of the plasmon resonances supported by the 1D nanotrench by calculating the electromagnetic local density of states (LDOS) at the center of the nanotrench opening using a two-dimensional version of the boundary-element method (BEM) that exploits the translational invariance of the system along the y direction [8].

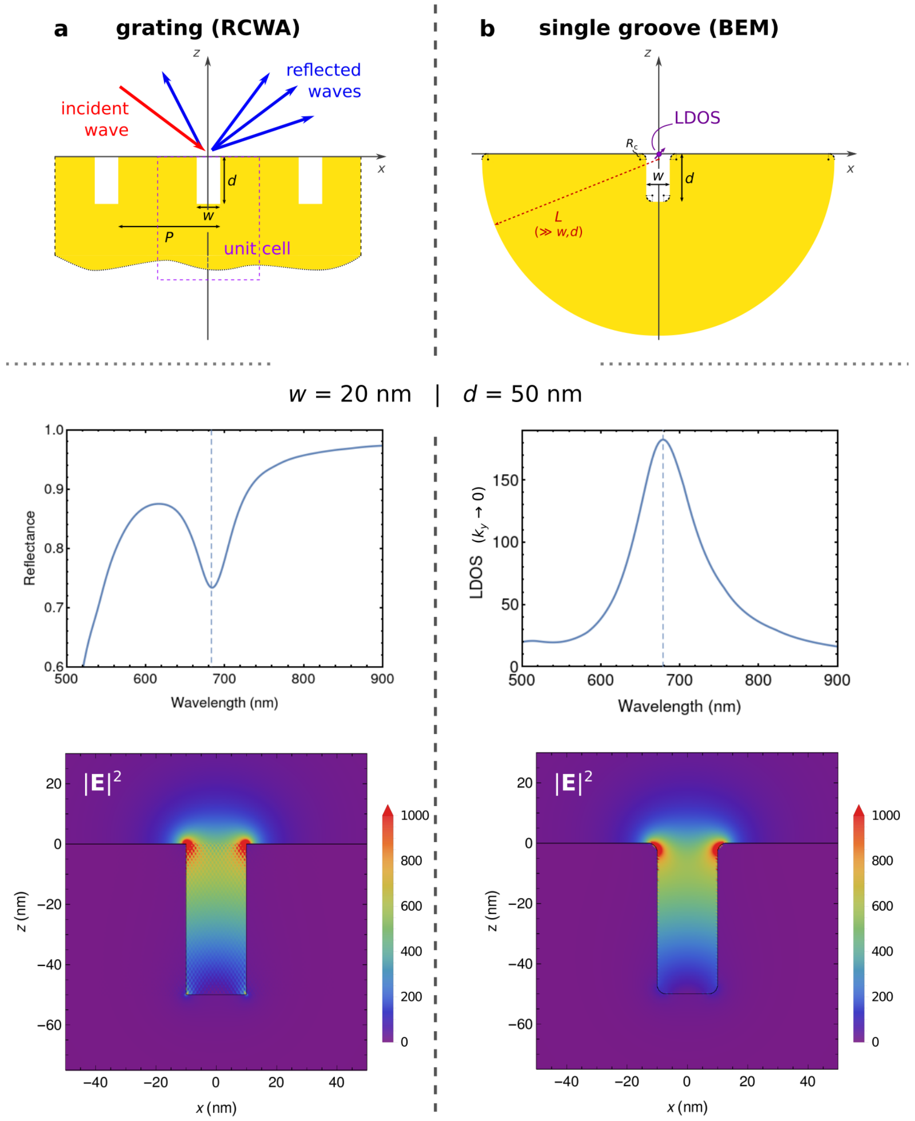

Fig. S7. Employed theoretical approaches for modeling the plasmonic response of gold nanotrenches. **(a)** Grating (period P) of gold nanotrenches of width w and depth d. The optical response of the system is calculated semi-analytically using the RCWA (top left), from which the reflectance (center left) and total $|\mathbf{E}|^2$ (bottom left) are obtained. **(b)** Isolated nanotrench on a gold substrate (top right). The structure is modeled using the BEM, where the (infinite) substrate is mimicked by a semi-circle of radius $L \gg w, d$. All corners are rounded with quarter-circles of radius $R_c = 3\,$nm. Plasmon resonances are revealed by peaks in the k_y-resolved LDOS evaluated at the center of the nanotrench opening (center right). In both methods, $|\mathbf{E}|^2$ has been normalized to the incident plane-wave (with $\mathbf{k}_{\rm inc} \parallel -\hat{\mathbf{z}}$ and $\mathbf{E}_{\rm inc} \parallel \hat{\mathbf{x}}$) field intensity, and its wavelength is selected to coincide with the plasmon resonance identified in the central panels.

Figure S7 shows an example of the plasmonic response (namely, the plasmon resonance wavelength and the associated electric-field distribution) obtained with each of these methods. Further details are provided in Secs. S3.A and S3.B below.

A. Modeling of plasmon modes in gold nanotrench using BEM

We compute the k_y-resolved LDOS[1] (as the system exhibits translation invariance along the y direction) at the center of the nanotrench opening (see Fig. S7b). From this quantity, the dispersion relation of the optical modes supported by the system is identifiable as the regions in the (k_y, ω)-space featuring an increased LDOS, as show in Figs. S8 and S9 below. In particular, Fig. S8 shows the LDOS for an electric field projected along the Cartesian directions. Notice that, while y- and z-projected LDOS are only enhanced in the vicinity of the light-line and of the high-momentum asymptote of the surface plasmon polariton (SPP) of a flat Au–air interface (green and white dashed lines, respectively), the x-projected LDOS exhibits a clear mode at small k_y with a pronounced LDOS enhancement (note the differences in the colorbar scales), which corresponds to the sought-after plasmon mode of the nanotrench. Figure S9a shows the total LDOS (i.e., the sum of the components shown in Fig. S8) in the region of interest, whereas Fig. S9b depicts a cut of the same data for $k_y \to 0$, in which the position of the maximum should compare well with the reflection dip observed in far-field measurements when the incident beam is polarized across the nanotrench (x direction) and its wave vector lies in the xz plane.

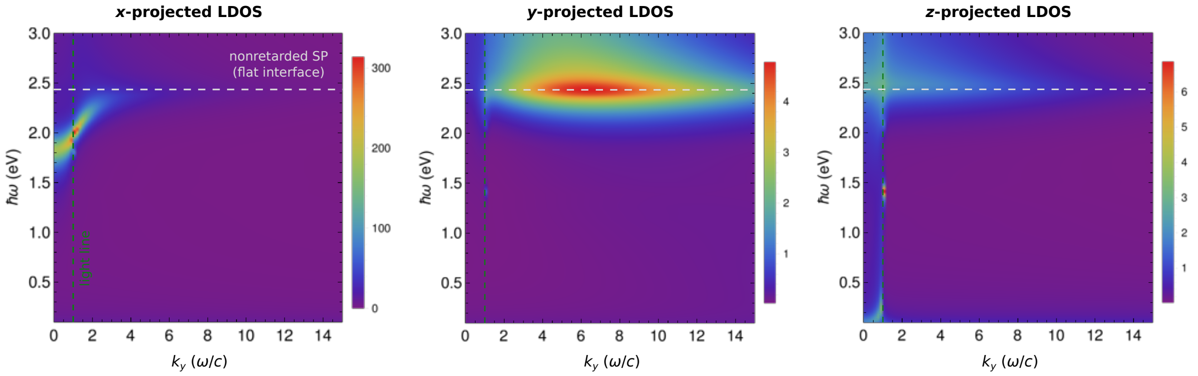

Fig. S8. Projections of the k_y-resolved LDOS (arb. units) evaluated at a point located at the opening of a gold nanotrench with $w = 20\,\mathrm{nm}$ and $d = 50\,\mathrm{nm}$, calculated using the BEM. Parameters: $R_c = 3\,\mathrm{nm}$ and $L = 5d$.

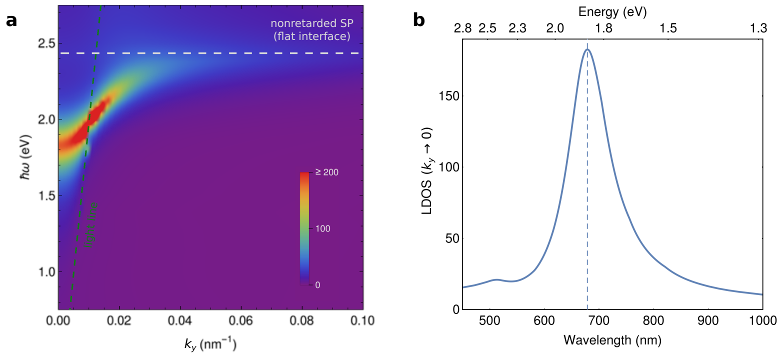

Fig. S9. (**a**) Dispersion relation via the total LDOS (arb. units) evaluated at a point located at the opening of a gold nanotrench, calculated using the BEM. (**b**) Cut for $k_y \to 0$ showing a large peak associated with the plasmon resonance localized at the opening of the gold nanotrench (the shoulder at smaller wavelengths is due to the surface plasmon mode of the flat Au–air interface, which can be launched when illuminating the groove). Parameters: Same as in Fig. S8.

---

[1] The k_y-resolved LDOS is defined here as $\mathrm{dLDOS}/\mathrm{d}k_y$, from which the total LDOS follows from $\int \frac{\mathrm{dLDOS}}{\mathrm{d}k_y}\,\mathrm{d}k_y$. Nevertheless, it should be noted that throughout this work we refer to the k_y-resolved LDOS simply as "LDOS" to simplify the language.

B. Plasmonic resonances in arrays of gold nanotrenches: Grating modes and channel resonances

Although here we are interested in the plasmon resonances localized within a single nanontrench, the samples are actually 1D periodic arrays of many trenches forming a grating. One of the most prominent methods to describe electromagnetic scattering of light off a grating is the RCWA [5, 6, 9], which not only accounts for the single-trench resonances but also for diffraction modes. The latter are know to exist since the early works of Wood, Rayleigh, and Fano [10, 11, 12, 13] and are now well understood [14, 15, 16]. In particular, the so-called Wood anomalies correspond to diffracted orders scattered at grazing angles and occur at wavelengths that satisfy the condition $\lambda_n^{(\pm),\mathrm{W}} = \frac{P}{n}(\pm 1 - \sin\theta_\mathrm{i})$ for the the n-th diffracted order; notice that these are *independent* of the material. Focusing on TM modes, another type of grating mode is the grating-assisted excitation of SPPs whereby the periodic structure endows in-plane momentum in packs of $n\frac{2\pi}{P}$, and thus the scattered waves carrying in-plane momentum $q_n = k_0 \sin\theta_\mathrm{i} + n\frac{2\pi}{P}$ can excite Bloch-type SPP modes whenever $\mathrm{Re}\{q_\mathrm{SPP}\} \simeq \left|k_0 \sin\theta_\mathrm{i} + n\frac{2\pi}{P}\right|$, where $k_0 \equiv \omega/c$ and $q_\mathrm{SPP} = \frac{\omega}{c}\sqrt{\frac{\epsilon_\mathrm{m}}{\epsilon_\mathrm{m}+1}}$ is the dispersion of SPPs at a flat air–metal interface.

Below, we focus on two cases: the one we are interested, i.e., in which the plasmon resonance associated with an individual nanotrench is spectrally well-separated from the grating modes, and another one where both the diffraction grating modes and the single-trench resonances coexist and interact.

a. Spectrally isolated single-trench plasmon resonance For certain sets of parameters $\{P, \theta_\mathrm{i}, w, d\}$, the plasmon resonance associated with an individual nanotrench can be well isolated, within a relatively large spectral range, from grating-assisted resonances. Such an example is presented in Fig. S10, showing the reflection of a normally incident plane-wave from an array of $w = 20$ nm and $d = 50$ nm nanotrenches with period $P = 1000$ nm carved on a gold substrate (as in Fig. S7a). Within the spectral window depicted in the figure, a clear reflection dip signals the

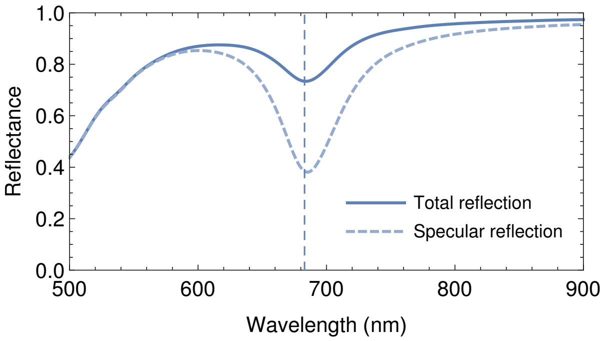

Fig. S10. Reflectance of a TM-polarized ($\mathbf{E}_\mathrm{inc}$ polarized across the nanotrenches' width) plane-wave incident on a 1D gold grating at an angle $\theta_\mathrm{i} = 0$, showing both the total (all scattered angles collected) and the specular (contribution of the reflected angle for which $\theta_\mathrm{ref} = \theta_\mathrm{i}$) reflection. Parameters: $w = 20$ nm, $d = 50$ nm, and $P = 1000$ nm.

presence of a resonance, which in this case corresponds to the plasmon mode of the individual nanotrenches. We emphasize that the obtained resonant wavelength indeed compares well with the single-trench resonance previously calculated with BEM (cf. Fig. S9b).

b. Coexistence of single-nanotrench and diffraction-grating modes We now consider different sets of parameters $\{P, \theta_\mathrm{i}, w, d\}$ and extend the wavelength range under analysis, and interpret the different modes imprinted in the reflectance spectrum from 1D arrays of gold nanotrenches. Some representative spectra are shown in Fig. S11, for different configurations. In contrast to the spectrum illustrated in Fig. S10, each spectrum now exhibits a number of features corresponding to the excitation of plasmons of the individual nanotrenches as well as the grating modes mentioned earlier. Selecting, for the sake of clarity, two of those spectra, we reproduce them in Fig. S12 along with the identification of the observed spectral features. Besides the broader single-trench plasmon resonance at around 685 nm, the spectra also exhibits a number of grating modes (Wood anomalies and grating-excited SPPs modes); see the figure caption for details.

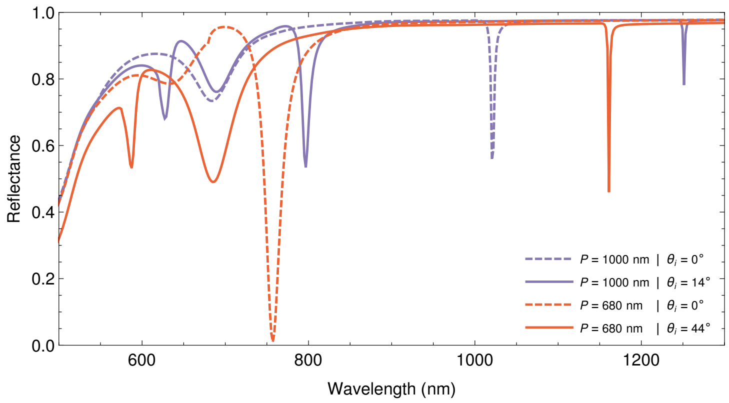

Fig. S11. Reflectance spectra, obtained through RCWA calculations, of periodic nanotrenches carved on a gold substrate. The incident plane wave is TM-polarized (i.e., with the electric field parallel across the trenches) and the nanotrenches' dimensions are $w = 20\,\mathrm{nm}$ and $d = 50\,\mathrm{nm}$ (the remaining parameters are indicated in the figure).

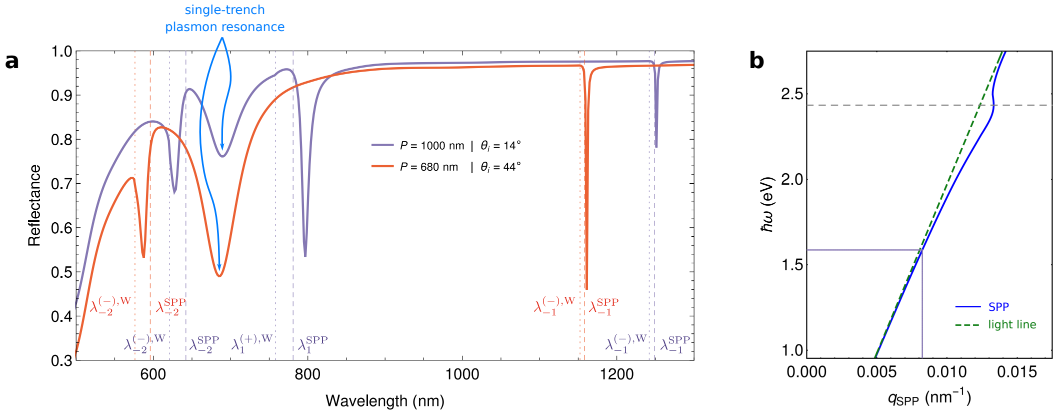

Fig. S12. **(a)** Identification of the spectral features exhibited in the spectra shown in solid lines in Fig. S11. Besides the single-trench plasmon resonance at around 685 nm, the spectra also display a number of grating modes: Wood anomalies, at $\lambda_n^{(\pm),\mathrm{W}} = \frac{P}{n}(\pm 1 - \sin\theta_\mathrm{i})$, indicated as vertical dotted lines, and grating-excited SPP resonances, approximately located at the wavelengths where $|q_n| = |k_0 \sin\theta_\mathrm{i} + n\frac{2\pi}{P}|$ intersects the SPP dispersion [see panel (b)], marked as dashed vertical lines (differences can be attributed to the presence of losses and interactions beyond the empty lattice approximation, $\mathrm{Re}\{q_\mathrm{SPP}\} = |q_n|$). **(b)** Dispersion of SPPs sustained at a flat air–gold interface (the horizontal dashed line indicates the high-momentum SPP energy asymptote.

C. Characterization of the plasmon resonance in a gold nanotrench

As remarked above—in Secs. S3.A and S3.B—the spectral properties of the plasmon mode associated with an individual nanotrench can be well-described by both the BEM and RCWA techniques (provided that in the latter the single-trench resonance is well-isolated from the grating modes), as a comparison between Figs. S10 and S9b suggests (for a nanotrench with width $w = 20\,\mathrm{nm}$ and depth $d = 50\,\mathrm{nm}$). In Fig. S13, we offer additional BEM and RCWA results, covering a substantial range of geometrical parameters $\{w, d\}$ of the nanotrenches, which further corroborates this conclusion.

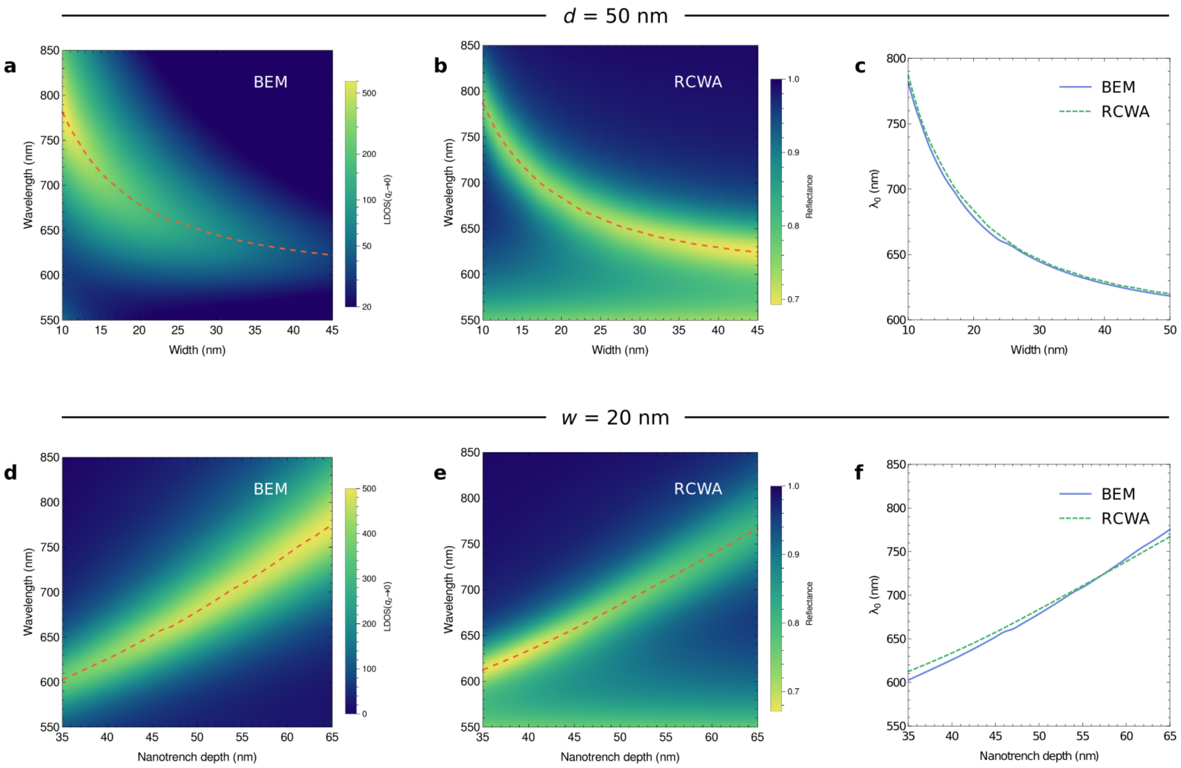

Fig. S13. Dependence of the single-trench plasmon resonance on the geometry of the nanotrench (width w and depth d), computed with **(a–b)** the BEM and **(d–e)** via RCWA. In the BEM calculations, we set $R_c = 3\,$nm and $L = 250\,$nm, whereas in RCWA calculations we take $P = 1000\,$nm and $\theta_i = 0$.

S4. TUNING THE NANOTRENCH PLASMON RESONANCE: INFLUENCE OF ALD COATING AND TRENCH SHAPE

A. Experiment

The theoretical analysis presented in Sec. S3 provides a comprehensive understanding of the optical response of the gold nanotrenches and establishes the necessary guidelines for designing our devices. In particular, we have shown that the plasmon resonance of an individual nanotrench can be tuned by tailoring its width.

To verify that the measured resonances indeed originates from plasmons associated with the individual nanotrenches (and thus free from the influence of grating modes), we prepared two sets of samples with pitch sizes of $P = 1000\,$nm and $P = 680\,$nm. These were fabricated in the same wafer to ensure uniformity in the gold thickness and HSQ-processing parameters, including dose and designed widths. To determine the resonance wavelength, we employed objectives with different magnifications and numerical apertures (NAs): 0.25 NA (and 10×) and 0.7 NA (and 100×) for larger and smaller pitch sizes, respectively (Fig. S14a). This choice allowed us to effectively measure the far-field reflection from the limited TMD-covered region (typically tens of μm^2) using the 680 nm pitch size, leveraging the large magnification and tightly focused light spot of the high-NA objective. To determine the resonance wavelength, we conducted reflection measurements using a white light source, which was linearly polarized in the direction perpendicular to the trench, that is, along the x-axis (see Fig. 1a in the main text). The reflection signal from the region containing the nanotrench was then normalized to the signal obtained from a neighboring area without any patterning.

We estimated the width of the nanotrenches indirectly from scanning electron microscope (SEM) images of the corresponding HSQ lines (Fig. S14b) and found width variations around ±3 nm within the same array. The width-dependence of the plasmonic resonance is depicted in Fig. S14c–d, exhibiting a blueshift with increasing width. Notably, the overlapping resonance positions of the two sets of samples (Fig. S14d) confirm that the measured resonances indeed arise from plasmons of the individual nanotrenches and not from grating-assisted

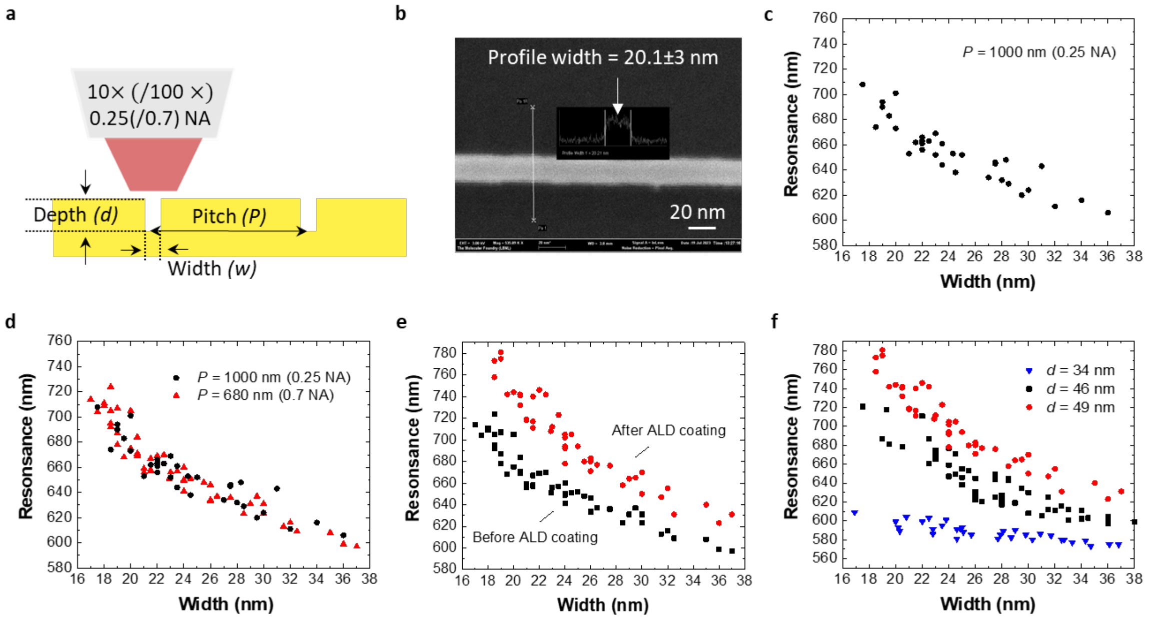

Fig. S14. Width-dependent plasmonic resonance of the nanotrenches. **(a)** Scheme of the setup. We employed objective lenses with NA = 0.7 and NA = 0.25 for the nanotrench arrays with P = 680 nm and P = 1000 nm, respectively. **(b)** SEM image of a HSQ line. The inset shows the vertical line cut indicated in the SEM image, from which the width of the nanotrench (i.e., the complementary of the HSQ line) is estimated. **(c)** Width-dependent plasmon resonance of the nanotrenches (d = 49 nm and P = 1000 nm) measured before ALD coating. **(d)** Width-dependent plasmon resonance of the nanotrenches of the two sets of samples (d = 49 nm) with different pitch size (prior to ALD coating). **(e)** Width-dependent plasmon resonance of the nanotrenches (d = 49 nm and P = 680 nm) before and after the 2 nm Al_2O_3 ALD coating. **(f)** Width-dependent plasmon resonance for nanotrenches with different depths (measured from the P = 680 nm sample), as indicated in the figure label.

modes. Moreover, we note that the resonance position shows similar variations (i.e., spread in wavelength for nominally equivalent widths) in the two cases, which results from commensurate variations in width (and shape) within the same nanotrench array.

The impact of the 2-nm-thick Al_2O_3 layer was investigated by comparing the width-dependent resonances before and after the ALD coating. As shown in Fig. S14e, the resonance positions redshift after ALD deposition as intuitively expected. This behavior is consistent with a previous report [17]. The resonance wavelengths of the ALD-coated sample serve in this work as the detuning wavelengths (with respect to the WSe_2 exciton) to determine the polariton dispersion curve.

Incidentally, we have also prepared nanotrenches with varying depths. As depicted in Fig. S14f, the plasmon resonances exhibit a redshift with increasing depth, as anticipated by the theoretical calculations presented above (Fig. S13d–f).

B. Theory: Effect of the nanotrench shape

Thus far, we have assumed in the theoretical calculations that the cross sections of the trenches are rectangular-like, as in the nominal lithography design. However, a comparison between experiment data and theoretical calculations suggests that the nanotrenches have slanted walls.

Figure S15 shows the width-dependent plasmon resonances for different nanotrench shapes, namely, the idealized box-shape as well as those with U- and V-shapes, contrasting experimental data (symbols) with theoretical calculations (lines); see Fig. S16 for geometric details. In the latter, we have considered few-nanometer variations of the trench depth around the experimentally estimated value of $d_{AFM} \approx 49$ nm to accommodate for the uncertainty in the measurement. The comparison between the experimental data and the theoretical calculations suggests that the nanotrenches have V-shape cross sections, which is compatible with previously reported experimental observations

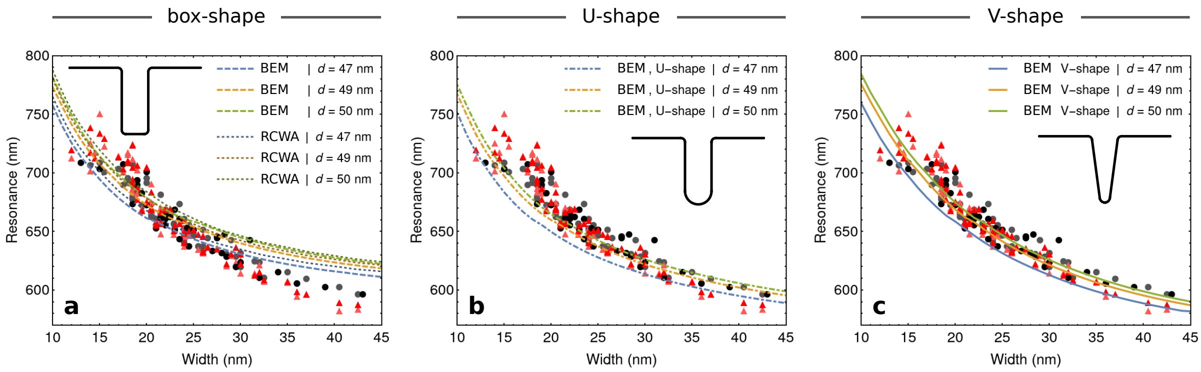

Fig. S15. Plasmon resonance wavelength in gold nanotrenches (without ALD coating) as a function of the width, for selected groove depths (see labels) and different shapes: **(a)** box-like, **(b)** U-shape, and **(c)** V-shape; see Fig. S16 for details on the geometry. The symbols correspond to experimental data (from a sample with $P = 680\,\text{nm}$) and the curves to theoretical calculations (see labels). The trench depth, estimated from AFM measurements of the HSQ lines, is $d_{\text{AFM}} \approx 49\,\text{nm}$ (with a few-nm of uncertainty).

where similar nanofabrication techniques have been employed [17, 18, 19] (cf. Fig. S17). We note, nonetheless, that both the "standard" box-like-shape and the V-shape yield similar results in the $w \in [10, 30]$ range.

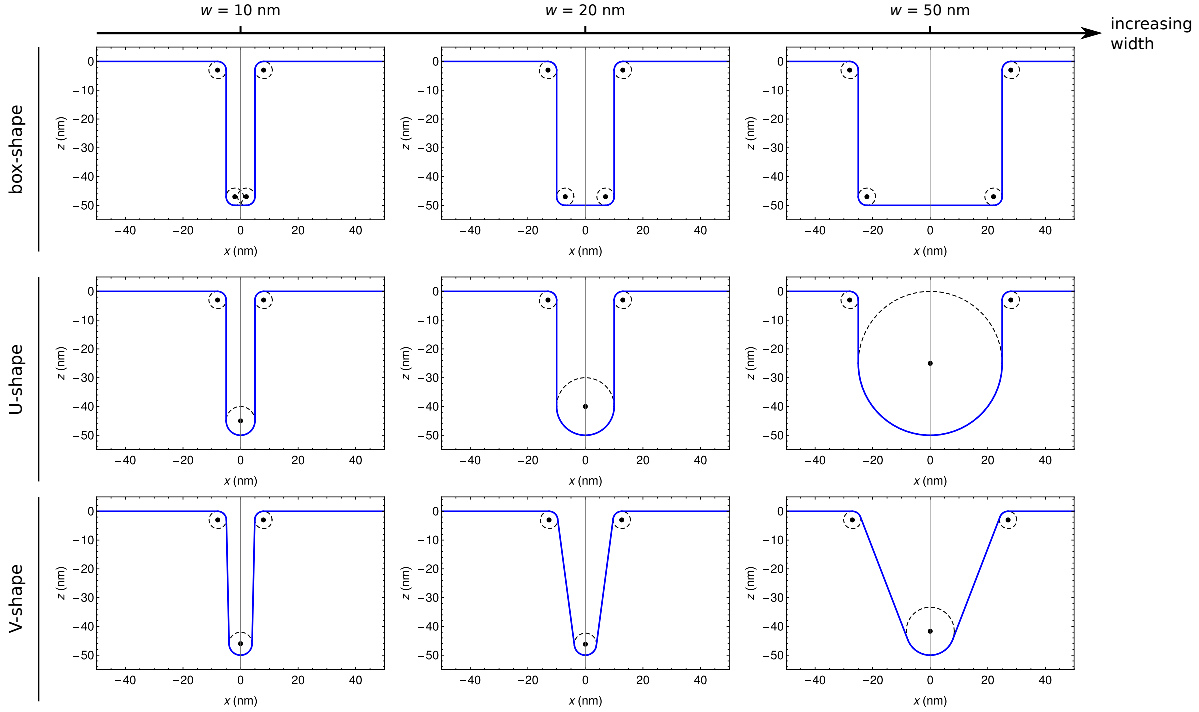

Fig. S16. Construction of different groove shapes. For the "standard" box-like shape, all corners are rounded with a radius of curvature R_c (top row). For the U-like shape, the top corners are also rounded with quarter-circles of radius R_c, but the trench's bottom is taken as a semi-circumference of radius $R_b = w/2$. For the V-like shape, the top corners are smoothed with a radius of curvature R_c, whereas the trenches bottom is rounded by a section of a circumference with width-dependent radius $R_b(w) = [4 - (20 - w/6)/4]w/10 + (20 - w/6)/4$. This $R_b(w)$ has been chosen *ad hoc*, in such as way that the resulting shapes reasonably reproduce to the ones reported in previous studies (see Fig. S17).

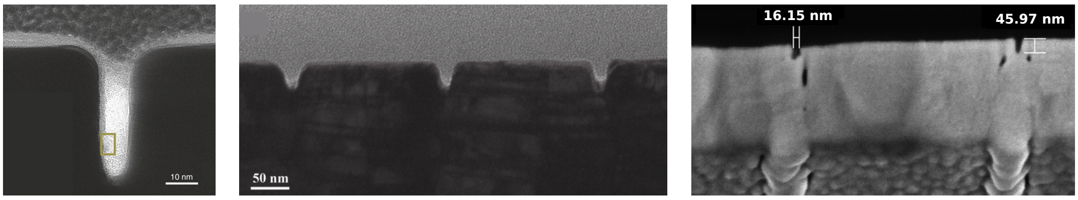

Polyakov et al., *Sci. Rep.* **2**, 933 (2012) | Polyakov et al., *Appl. Phys. Lett.* **98**, 203104 (2011) | Polyakov et al., *JVST B.* **29**, 06FF01 (2011)

Fig. S17. Experimental images of gold nanotrenches reported in the literature, fabricated using a similar procedure as the one employed in this work. Adapted from Refs. [17, 18, 19], as indicated below each image.

C. Influence of the ALD coating

Figure S18a compares theoretical and experimental results of the pre- and post- ALD-coated samples (assuming a conformal 2-nm-thick Al_2O_3 ALD coating). We perform calculations assuming depths ±2 nm around the one estimated from AFM measurements of the HSQ lines and choose the value of d that better fits the experimental data. Figure S18b shows a similar analysis but for three distinct nanotrench depths (the same as in Fig. S14f).

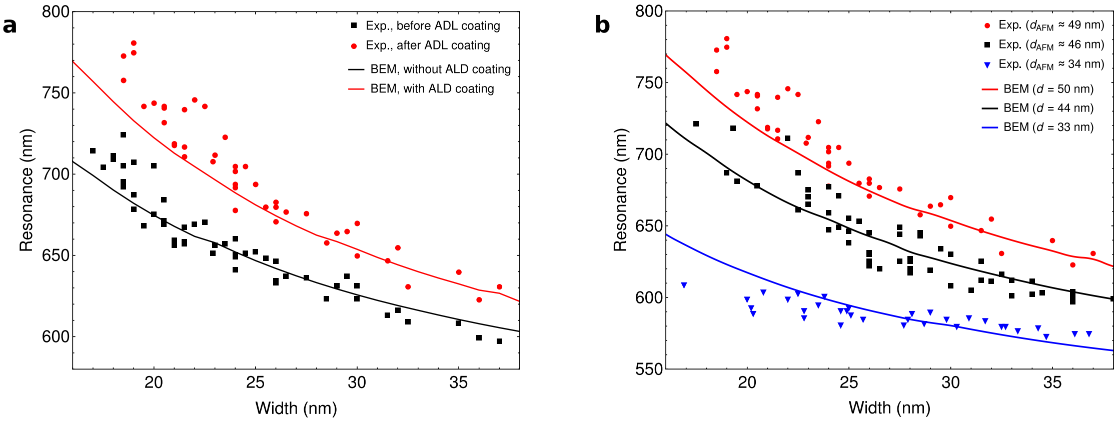

Fig. S18. Plasmon resonance wavelength in gold nanotrenches as a function of width. Symbols correspond to experimental measurements and solid lines to BEM calculations (assuming V-shape trenches). **(a)** Plasmon resonances before and after Al_2O_3-coating (shown in black and red, respectively). The experimentally estimated depth, obtained through AFM measurements of the HSQ lines, is $d_{AFM} \approx 49$ nm, while $d = 50$ nm is the best fit to the data from BEM calculations (note that $d_{AFM} \approx d$, well within the uncertainty in the AFM measurement). **(b)** Plasmon resonance in Al_2O_3-coated gold nanotrenches as a function of the width, for nanotrenches of different depths: see labels for the AFM-estimated measurements and corresponding best fit of d in the BEM calculations (note that $d_{AFM} \approx d$, well within the uncertainty of the AFM measurement). In both panels, the Al_2O_3-layer has a thickness of $t_{ALD} = 2$ nm and a relative permittivity $\epsilon_{Al_2O_3} = 2.6$ [20].

D. Polarization dependence

Figure S19a shows polarization-dependent reflection measurements from ALD-coated gold nanotrenches (i.e., in a region uncovered by WSe_2, adjacent to the location where the data in Fig. 1d were obtained). At 0° polarization, a single resonance dip is observed in the spectrum, progressively diminishing as the polarization angle is rotated toward 90°. This attests that nanotrench plasmon resonance is strongly polarized across the trench width, as already anticipated in Fig. S8, and further corroborated by the RCWA calculations shown in Fig. S19b.

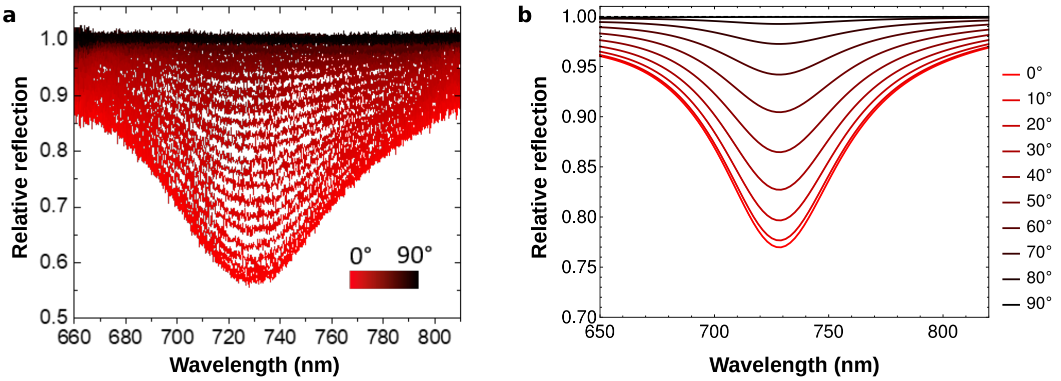

Fig. S19. Relative reflection from ALD-coated nanotrenches (without WSe_2) for varying polarization angles, from 0° (perpendicular to the trench) to 0° (along the trench). **(a)** Experimental data. **(b)** RCWA calculation (taking $P = 1000$ nm), from a ALD-coated gold nanotrench ($w = 19$ nm and $d = 50$ nm) assuming a normally incident plane-wave with different polarization angles (see labels).

E. Impact of PDMS residues

PMDS is known to leave residues following the dry transfer process [1]. The presence of PDMS residues is visually confirmed through the camera view (Fig. S20a) where the areas in contact with the PDMS during the transfer process appear lighter. These residues do not significantly affect the coupling of the trench plasmon to the TMD excitons because the PDMS leftovers are situated above the WSe_2 monolayer (Fig. S20b), and not between the trench and the TMD. Although these residues are very small (hardly detectable in AFM), they still induce a slight

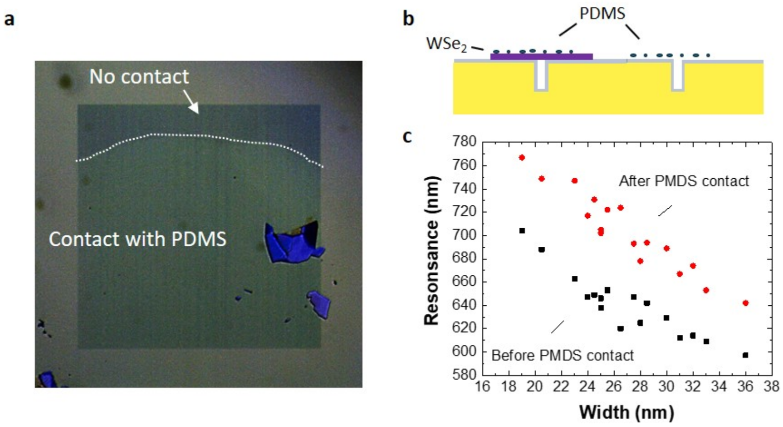

Fig. S20. Impact of PDMS residues. **(a)** Camera view of the sample after the transfer process. The dotted white line marks the border of the area contaminated with PDMS residues. **(b)** Schematic view illustrating the PDMS residues, in the TMD-covered region as well as in the area adjacent to it. **(c)** Plasmon resonances as a function of the nanotrench width (for $d_{AFM} = 46$ nm trenches) before and after contact with the PMDS.

change in the dielectric environment, which, although minor, influences the plasmon resonance of the nanotrench as it is extremely sensitive to the optical constants of the nearby media. This was also observed in areas immediately adjacent to the WSe_2 layer.

Figure S20c presents a comparison of the width-dependent plasmon resonances of the nanotrenches before and after coming into contact with the PDMS, showing a consistent redshift of $\approx 60\,\mathrm{nm}$. Since the dielectric constant of PDMS is known to vary considerably (e.g., depending on the processing conditions, etc.) [21] and PDMS residues are neither continuous nor uniform, in our theoretical calculations we take this effect into account by adjusting the depth of the nanotrenches so that it reproduces the observed PDMS-induced redshift. Specifically, in the theoretical calculations presented in the main text (namely, Figs. 2d, 3c, and 4a–b) we have used $d = 50\,\mathrm{nm}$ instead of the experimentally estimated $d_{\mathrm{AFM}} = 46\,\mathrm{nm}$, wherein the 4 nm depth difference effectively compensates for the slight resonance wavelength offset due to the presence of PDMS residues.

S5. PLASMON–EXCITON COUPLING IN WSe_2-COVERED GOLD NANOTRENCHES

Figure S21 shows spectra exhibiting energy splittings associated with strong coupling between excitons in the WSe_2 monolayer and plasmons in the gold nanotrenches under near nearly zero-detuning conditions (i.e., when the plasmon resonance matches that of the TMD exciton).

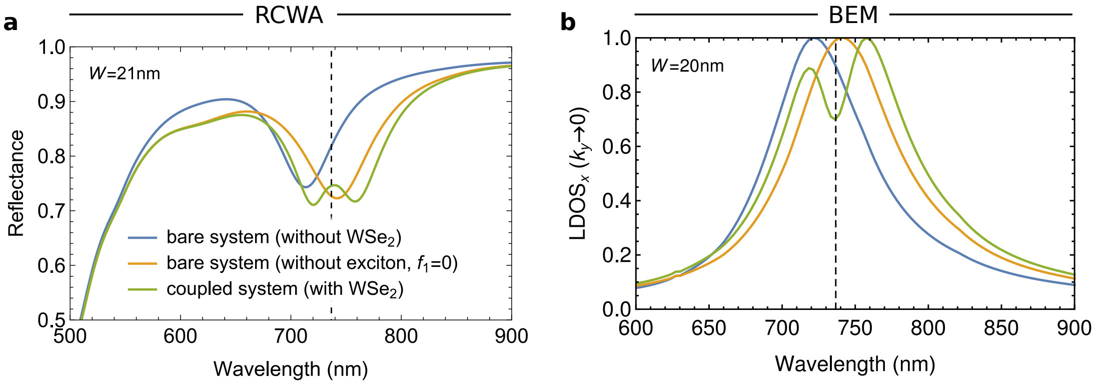

Fig. S21. Optical response of Al_2O_3-coated Au nanotrenches (d = 50 nm) with (green curves) and without WSe_2 (blue curve). The orange curve takes into account the presence of the high background permittivity of the WSe_2 but without the excitonic resonance [setting $f_1 = 0$ in Eq. (S1)], whose effect is to redshift the "bare" plasmon resonance. **(a)** Reflectance calculated using the RCWA method (assuming box-like-shaped trenches). **(b)** Computed $\mathrm{LDOS}_x(k_y \to 0)$, evaluated at $z = -5$ nm, obtained using the BEM (assuming V-shaped trenches).

A. Plasmon–exciton dispersion diagram and coupled-oscillator model fit

As shown in Sec. S3, the plasmon resonance of the gold nanotrench can be tailored by changing the trench geometry, and, in particular, its width. In Figs. S22 and S23, we present the plasmon–exciton dispersion diagrams obtained by sweeping the nanotrench plasmon resonance across the bare excitonic resonance (at $E_{\mathrm{exc}} = \hbar\omega_{\mathrm{exc}} = 1.683$ eV or $\lambda_{\mathrm{exc}} = 736.7$ nm), showing a clear anticrossing behavior characteristic of strong coupling [22, 23].

We quantify the plasmon–exciton coupling strength by fitting the spectral features associated with the lower and upper polaritons (LP and UP, respectively) to the coupled-oscillator model (COM) [22, 23], namely (neglecting losses)

$$E_{\pm}(w) = \frac{E_{\mathrm{p}}^{*}(w) + E_{\mathrm{exc}}}{2} \pm \frac{1}{2}\sqrt{4g^2 + [E_{\mathrm{p}}^{*}(w) - E_{\mathrm{exc}}]^2}, \qquad \text{(S2)}$$

where $E_+ \equiv E_{\mathrm{UP}}$ and $E_- \equiv E_{\mathrm{LP}}$ denote the energies of the UP and the LP, respectively, and g is the plasmon–exciton coupling strength. The corresponding Rabi-like splitting is thus $\Omega = 2g$. Here, $E_{\mathrm{p}}^{*}(w)$ denotes the width-dependent plasmon energy of the nanotrench *including* the effect of the non-resonant, "background" permittivity of WSe_2 in the absence of the bright A exciton [i.e., with $f_1 = 0$ in Eq. (S1)], which further redshifts the nanotrench plasmon energy (see Fig. S21).

It should be noted that in the main text we discuss the results using E_{p} as the "detuning wavelength" as E_{p}^{*} can not be directly measured. In particular, in Fig. 2d, we take the x-axis as $\lambda_{\mathrm{p}}(w) = 2\pi\hbar c/E_{\mathrm{p}}(w)$ instead of $\lambda_{\mathrm{p}}^{*}(w) = 2\pi\hbar c/E_{\mathrm{p}}^{*}(w)$, where $E_{\mathrm{p}}^{*}(w) \approx E_{\mathrm{p}}(w) - \Delta E$ and $\Delta E \approx 44.8$ meV is the (mean) shift between the "w/o TMD" and "w/o exciton ($f_1 = 0$)" lines in Figs. S22 and S23.

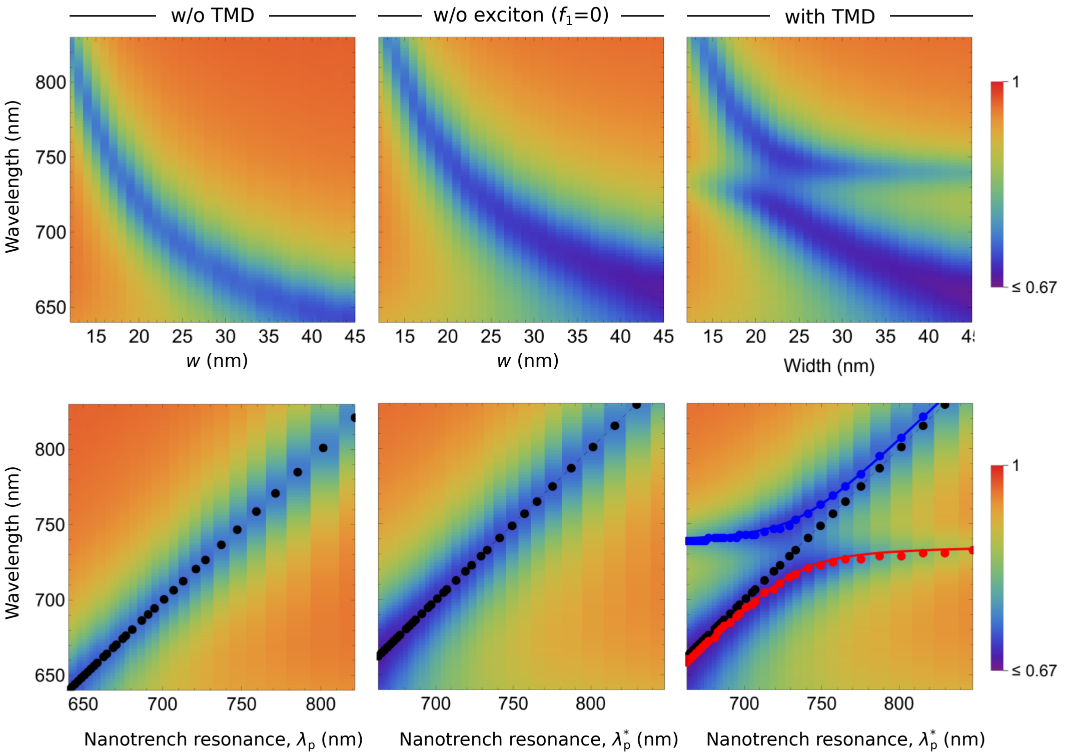

Fig. S22. Same as in Fig. S21a, for varying width. The lower-right panel also shows the coupled-oscillator model fit, with an energy splitting of $\Omega = 2g = 81.7\,\mathrm{meV}$.

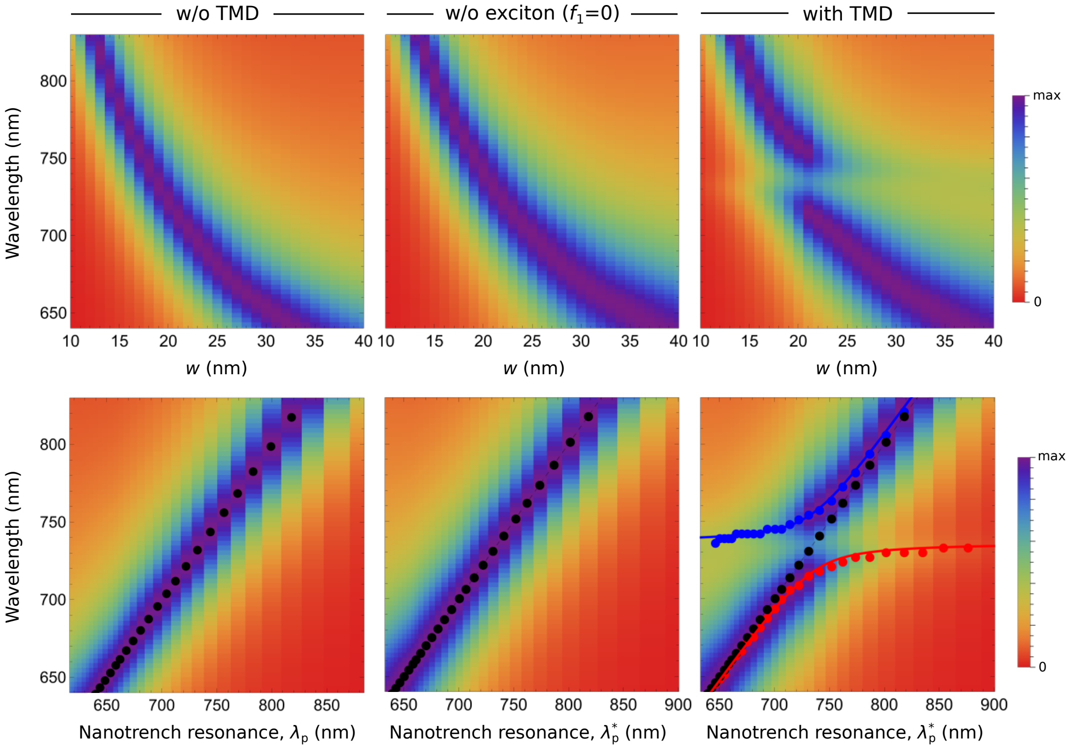

Fig. S23. Same as in Fig. S21b, for varying width. The lower-right panel also shows the coupled-oscillator model fit, with an energy splitting of $\Omega = 2g = 86.6\,\mathrm{meV}$.

1. *Influence of the uncoupled exciton background on the determination of the Rabi-like splitting*

While the plexciton absorption signal is substantially stronger than that of the bare, uncoupled exciton (≈ 40% versus ≈ 3%, respectively; see in Fig. 1d in the main text), the latter does contribute as a background affecting the determination of the Rabi-like splitting (specially since the area between trenches is much larger than the trench width, signal that is also collected during our far-field reflection measurement).

Figure S24 shows the reflection spectra, obatined using the RCWA method, for a zero-detuned sample for two orthogonal polarizations [across (TM) and along (TE) the nanotrench] and for the R_{TM}/R_{TE} ratio. Notably, under TE illumination, no trench plasmon is excited, and the spectrum is indistinguishable from that of a flat, unpatterned WSe_2–Al_2O_3–Au structure ($R_{TE} = R_{flat}$). The nanotrench width, which defines the strongly coupled area, is much smaller (approximately 34 times) than the flat region. Therefore, by plotting R_{TM}/R_{TE}, we can effectively remove the contribution from the uncoupled exciton background. This analysis indicates—see level splittings indicated in Fig. S24—that our results above (e.g., in Fig. S22) underestimated the Rabi splitting by 10 meV due to this background, leading to a corresponding underestimation of the coupling strength amounting to 5 meV (since $g \simeq \Omega/2$). Although this effect is relatively small, it may be important to bring our plexciton device into the strong-coupling regime.

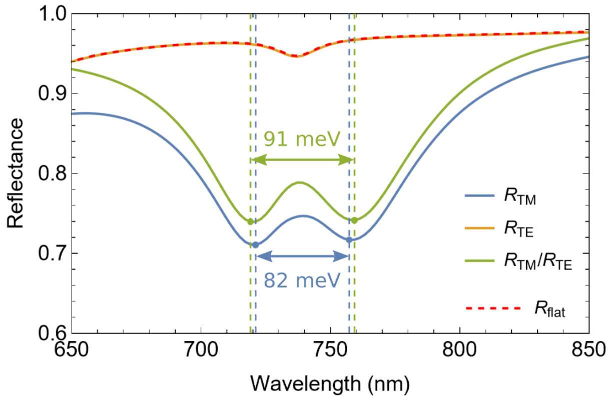

Fig. S24. Reflection spectra from a zero-detuned sample (solid lines), showing the spectra corresponding to TM and TE polarizations, along with the ratio R_{TM}/R_{TE} which effectively removes the uncoupled exciton background (see accompanying text). The arrows show the retrieved Rabi-like splitting base on both R_{TM} and R_{TM}/R_{TE}. The spectra were calculated using the RCWA for a normally illuminated sample consisting of an array (of period 1 μm) of 50-nm-deep nanotrenches.

B. Spatial localization of the lower polariton

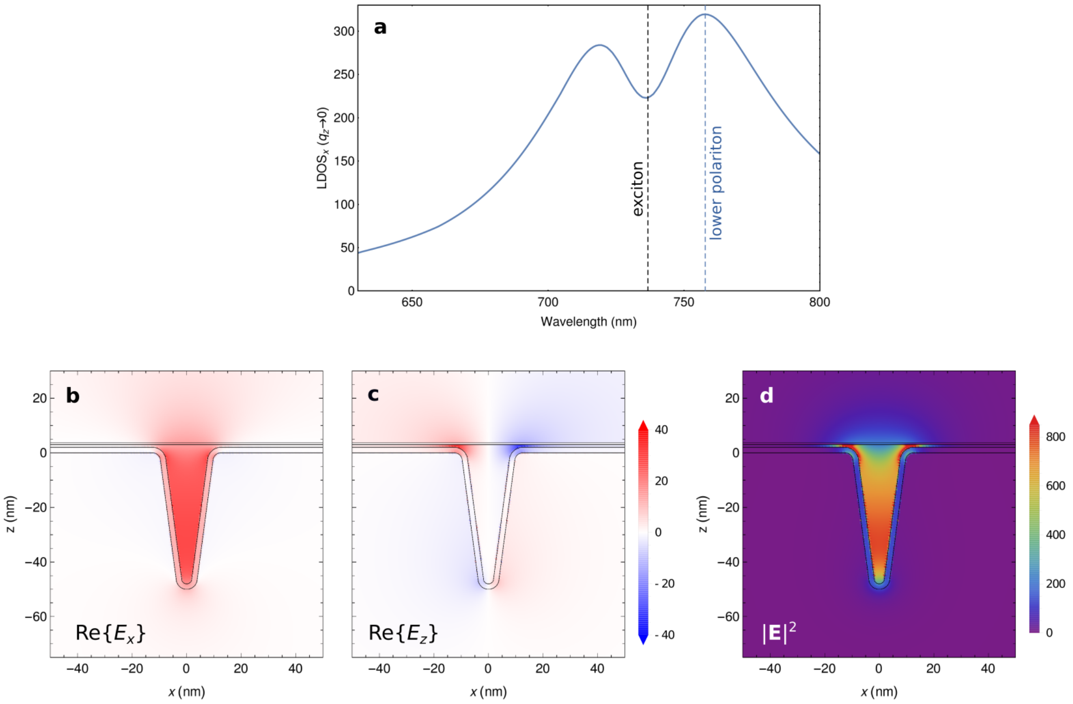

Fig. S25. Properties of the plexciton associated with the lower polariton branch calculated using the BEM. **(a)** x-projected LDOS in a WSe_2–Au-nanotrench system under zero-detuning conditions (same as in Fig. S21b) with the exciton and lower polariton resonance wavelengths indicated by vertical dashed lines. The resonance wavelength of the latter is 758 nm. **(b–d)** Electric field under plane-wave illumination at the plexciton resonance wavelength (758 nm), assuming normal incidence and polarization along the x direction.

S6. ADDITIONAL MEASUREMENTS

A. Reflection data from an off-resonance control sample and from a zero-detuned sample

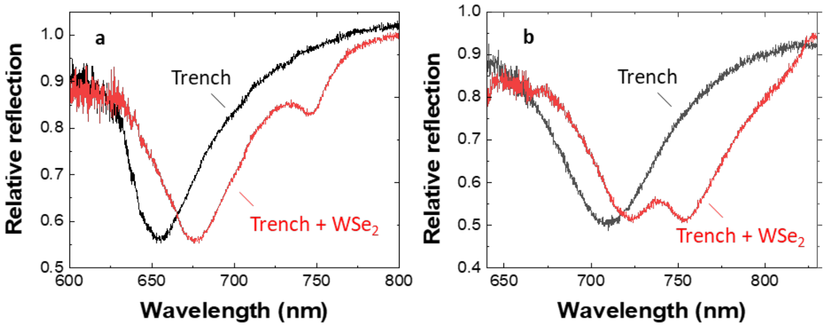

Fig. S26. Reflection measurements from of the **(a)** off-resonance and **(b)** on-resonance (zero-detuning) samples.

Figure S26 shows reflection measurements from the samples discussed in the paper (Fig. 3). Both off- (control) and on-resonance (at zero-detuning) samples have very similar widths ($w \approx 24$ nm), but different depths—around 34 nm and 46 nm, respectively—in order to tune the plasmon resonance of the nanotrench.

B. Line scans on different plexciton samples

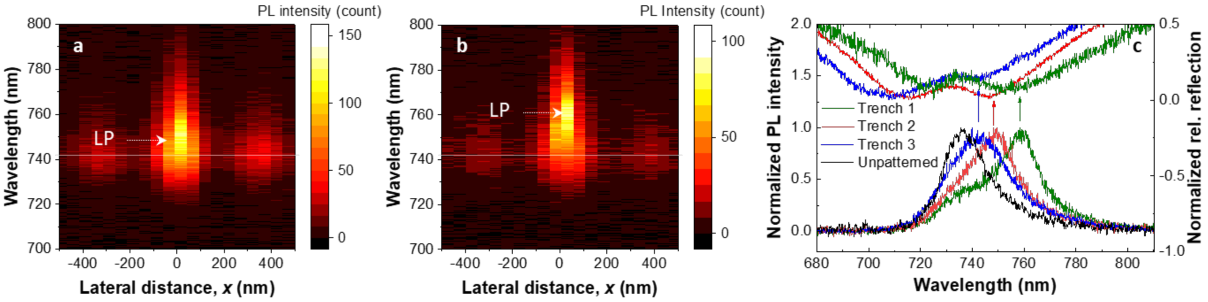

Fig. S27. Plexciton PL emission from a sample near zero detuning. **(a)** Line scan analysis of a plexciton sample with detuning resonance wavelength of 690 nm. The LP identified in the reflection spectrum is around 748 nm. **(b)** Line scan analysis of a plexciton sample with detuning resonance wavelength of 726 nm. The LP identified in the reflection spectrum is around 765 nm. **(c)** Normalized reflection and plexciton emission from samples with different detuning wavelengths.

C. Lorentzian peak fitting and polarization dependence of the plexciton PL emission

The PL spectra recorded with the pyramidal probe at the trench position is a mix of the plexciton emission signal and a background from the uncoupled and weakly coupled regions (the dielectric probe has spatial resolution just below the diffraction limit [4]). To remove the background in the polarization-dependent plexciton emission in Fig. 4d (main text), we fitted two Lorentzians to each spectra and polarization. We first obtained the center wavelength and linewidth of the bright exciton by fitting the uncoupled PL spectrum. We note that that variations in the peak wavelength and linewidth are minor and falls within the range of expected sample-to-sample variations. Then, for the plexciton PL emission spectra, we fixed the center wavelength and linewidth of the uncoupled exciton (first Lorentzian), leaving linewidth and the energy position of the second Lorentzian adjustable in the recorded

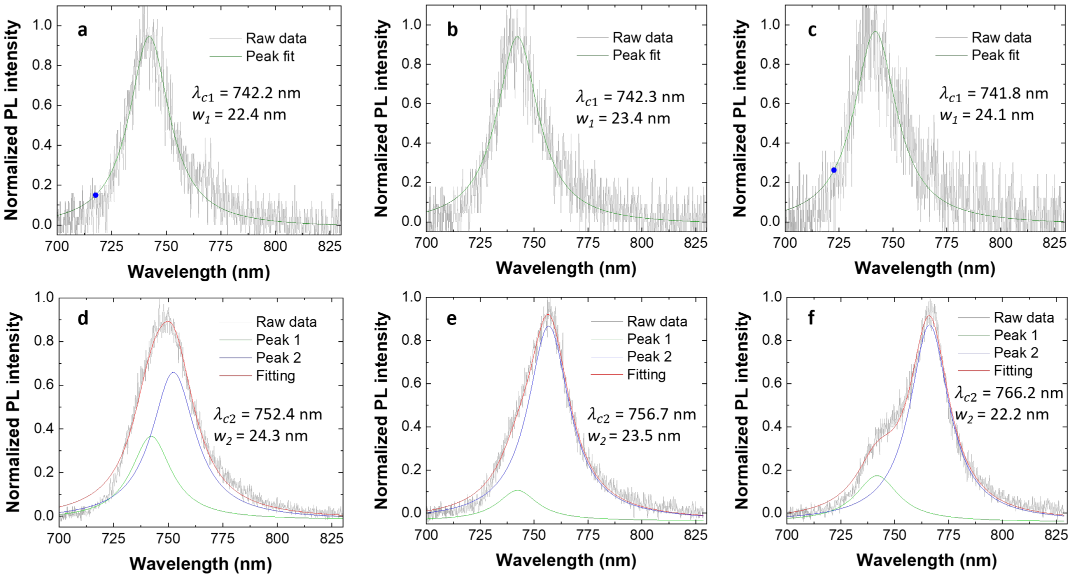

Fig. S28. Fit of plexciton PL emission from different samples, containing both uncoupled exciton and plexciton contributions. **(a–c)** Spectra acquired in the flat region between the trenches (representing PL from uncoupled exciton emission) and **(d–f)** at the nanotrenches from samples with detuning wavelength of 690 nm, 708 nm and 726 nm, respectively. Each spectrum in **(d–f)** is fitted with two Lorentzians, where the resonance and linewidth of one of them is kept constant [same as the ones obtained in **(a–c)**], and the other one is fitted with free parameters.

spectra at 0° polarization. The fitted linewidth and peak wavelength of the second Lorentzian is then kept fixed for the remaining fits (i.e., only the amplitude is adjusted).

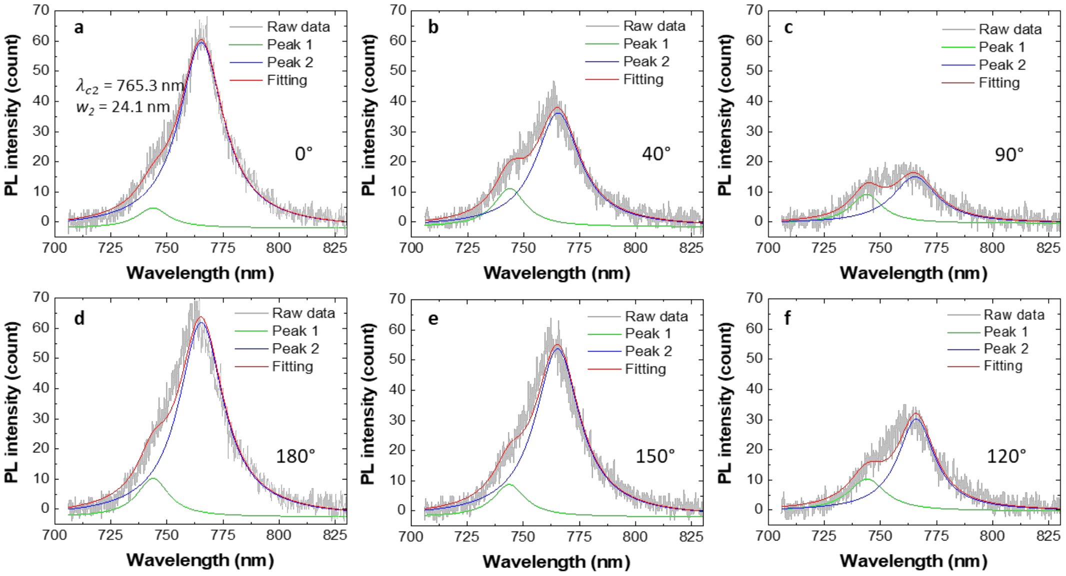

Fig. S29. Examples of PL spectrum fits acquired with a linear polarizer at different angles (see labels). **(a)** The spectrum for 0° polarization is fitted with two Lorentzians, where the resonance and linewidth of one of them is kept constant (same as the ones obtained in Fig. S28a, and the other one is fitted with free parameters. **(b–f)** Photoluminescence spectra for different polarizations (see labels), fitted with the parameters obtained in **a**.

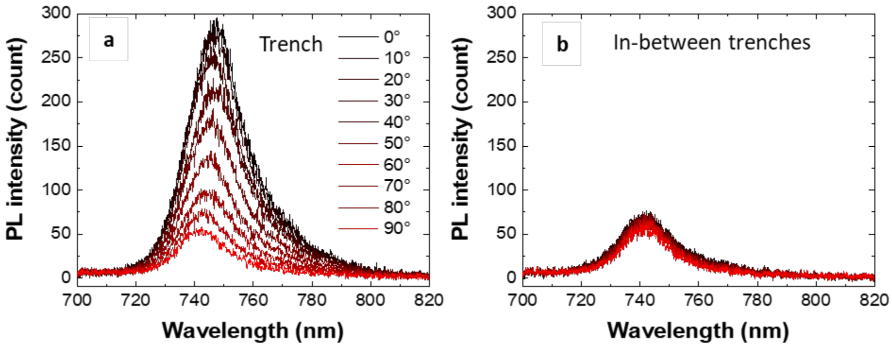

Fig. S30. Comparison of polarization states **(a)** of the plexciton and **(b)** of the bare, uncoupled exciton. The spectra in **a** were recorded at the trench position, whereas the spectra in **b** were recorded approximately 300 nm away from the trench (i.e., in the flat region between the nanotrenches).

SUPPLEMENTARY REFERENCES

[1] A. Castellanos-Gomez, M. Buscema, R. Molenaar, V. Singh, L. Janssen, H. S. J. van der Zant, and G. A. Steele, *Deterministic transfer of two-dimensional materials by all-dry viscoelastic stamping,* 2D Mater. **1**, 011002 (2014).

[2] L. H. Brixner, *X-ray study and thermoelectric properties of the* $W_xTa_{1-x}Se_2$ *system,* J. Electrochem. Soc. **110**, 289 (1963).

[3] W. T. Hicks, *Semiconducting behavior of substituted Tungsten Diselenide and its analogues,* J. Electrochem. Soc. **111**, 1058 (1964).

[4] J. Zhou, A. Gashi, F. Riminucci, B. Chang, E. Barnard, S. Cabrini, A. Weber-Bargioni, A. Schwartzberg, and K. Munechika, *Sharp, high numerical aperture (NA), nanoimprinted bare pyramid probe for optical mapping,* Rev. Sci. Instrum. **94**, 033902 (2023).

[5] M. G. Moharam, E. B. Grann, D. A. Pommet, and T. K. Gaylord, *Formulation for stable and efficient implementation of the rigorous coupled-wave analysis of binary gratings,* J. Opt. Soc. Am. A **12**, 1068 (1995).

[6] P. Lalanne and G. M. Morris, *Highly improved convergence of the coupled-wave method for TM polarization,* J. Opt. Soc. Am. A **13**, 779 (1996).

[7] J.-P. Hugonin and P. Lalanne, *RETICOLO software for grating analysis,* arXiv:2101.00901v3 (2023).

[8] F. J. García de Abajo and A. Howie, *Retarded field calculation of electron energy loss in inhomogeneous dielectrics,* Phys. Rev. B **65**, 115418 (2002).

[9] A. V. Lavrinenko, J. Lægsgaard, N. Gregersen, F. Schmidt, and T. Søndergaard, *Numerical Methods in Photonics*, 1st ed. (CRC Press, 2015).

[10] R. W. Wood, *On a remarkable case of uneven distribution of light in a diffraction grating spectrum,* Philos Mag. **4**, 396 (1902).

[11] Lord Rayleigh, *Note on the remarkable case of diffraction spectra described by Prof. Wood,* Philos. Mag. **14**, 60 (1907).

[12] Lord Rayleigh, *On the dynamical theory of gratings,* Proc. R. Soc. Lond. A **79**, 399 (1907).

[13] U. Fano, *The theory of anomalous diffraction gratings and of quasi-stationary waves on metallic surfaces (Sommerfeld's waves),* J. Opt. Soc. Am. **31**, 213 (1941).

[14] F. J. García de Abajo, *Colloquium: Light scattering by particle and hole arrays,* Rev. Mod. Phys. **79**, 1267 (2007).

[15] D. Maystre, Theory of Wood's anomalies, in *Plasmonics: From Basics to Advanced Topics*, edited by S. Enoch and N. Bonod (Springer, Berlin, 2012) pp. 39–83.

[16] A. A. Maradudin, I. Simonsen, J. Polanco, , and R. M. Fitzgerald, *Rayleigh and Wood anomalies in the diffraction of light from a perfectly conducting reflection grating,* J. Opt. **18**, 024004 (2016).

[17] A. Polyakov, K. F. Thompson, S. D. Dhuey, D. L. Olynick, S. Cabrini, P. J. Schuck, and H. A. Padmore, *Plasmon resonance tuning in metallic nanocavities,* Sci. Rep. **2**, 933 (2012).

[18] A. Polyakov, S. Cabrini, S. Dhuey, B. Harteneck, P. J. Schuck, and H. A. Padmore, *Plasmonic light trapping in nanostructured metal surfaces,* Appl. Phys. Lett. **98**, 203104 (2011).

[19] A. Polyakov, H. A. Padmore, X. Liang, S. Dhuey, B. Harteneck, J. P. Schuck, and S. Cabrini, *Light trapping in plasmonic nanocavities on metal surfaces,* J. Vac. Sci. Technol. B **29**, 06FF01 (2011).

[20] D. R. G. Mitchell, D. J. Attard, K. S. Finnie, G. Triani, C. J. Barbé, C. Depagne, and J. R. Bartlett, *TEM and ellipsometry studies of nanolaminate oxide films prepared using atomic layer deposition,* Appl. Surf. Sci. **243**, 265 (2005).

[21] J. E. Mark, *Polymer Data Handbook*, 2nd ed. (Oxford University Press, 2009).

[22] P. Törmä and W. L. Barnes, *Strong coupling between surface plasmon polaritons and emitters: a review,* Rep. Prog. Phys. **78**, 013901 (2014).

[23] P. A. D. Gonçalves, N. Stenger, J. D. Cox, N. A. Mortensen, and S. Xiao, *Strong light–matter interactions enabled by polaritons in atomically thin materials,* Adv. Optical Mater. **8**, 1901473 (2020).
